# MALDI-MS-based biomarker analysis of extracellular vesicles from human lung carcinoma cells†

**DOI:** 10.1039/d1ra04305f

**Published:** 2021-07-21

**Authors:** Zitong Yu, Chao Zhao, Shi Hu, Huitao Zhang, Wenbo Li, Renjie Zhang, Qian Luo, Hui Yang

**Affiliations:** Bionic Sensing and Intelligence Center, Institute of Biomedical and Health Engineering, Shenzhen Institute of Advanced Technology, Chinese Academy of Sciences Shenzhen 518055 China qian.luo@siat.ac.cn hui.yang@siat.ac.cn; Research Center for Medical Artificial Intelligence, Institute of Biomedical and Health Engineering, Shenzhen Institute of Advanced Technology, Chinese Academy of Sciences Shenzhen 518055 China; CAS Key Laboratory of Health Informatics, Shenzhen Institute of Advanced Technology, Chinese Academy of Sciences Shenzhen 518055 China

## Abstract

Extracellular vesicles (EVs) are actively secreted by mammalian cells. They are increasingly recognized as promising circulating biomarkers of disease progression. Matrix-assisted laser desorption/ionization-time of flight mass spectrometry (MALDI-TOF MS) is currently one of the most powerful techniques for the rapid analysis of biological samples, especially for discovering biomarkers for disease diagnosis and prognosis. It is unclear what cell culture medium components and EV isolation methods are suitable for MALDI-TOF MS analysis. Using a human lung carcinoma cell line (A549), we investigated and optimized the critical experimental conditions for EVs' protein profiling by combining differential ultracentrifugation and MALDI-TOF MS. The results demonstrated that medium components and ultracentrifugation procedures to extract EVs played important roles in MS detection. Compared with EV-depleted serum and normal serum medium, conditioned medium with 2% fetal bovine serum in this study maintained cell proliferation and displayed significant protein profiling of EVs. RPS27A (ribosomal protein), which plays an essential role in mRNA translation and ribosome assembly for the differentiation of cancer cells, was detected from the EVs of lung cancer cells associated with cancer cell migration and invasion. We also found the known tumor diagnosis marker, which is S100A10_S100 calcium-binding protein A10. Therefore, MALDI-TOF MS-based EV analysis with optimized experimental protocols can contribute to future development of rapid screening techniques of protein biomarkers associated with early cancer diagnosis.

## Abstract 2

The protein profile of extracellular vesicles isolated from human lung carcinoma cells has been detected by MALDI-TOF MS, which is essential for rapid discovery of cancer-related biomarkers.

## Graphical abstract

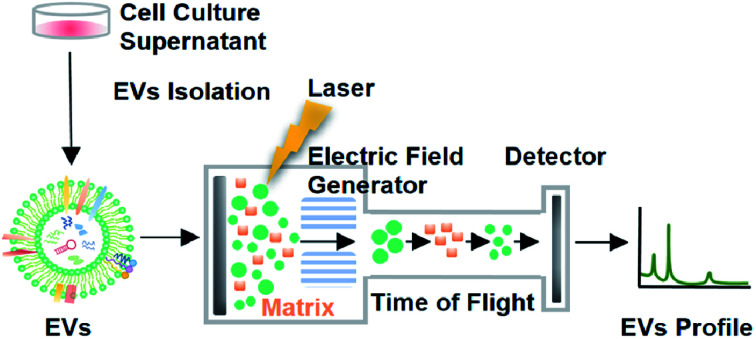

## Introduction

Extracellular vesicles (EVs) are heterogeneous, membrane-bound phospholipid vesicles that are actively secreted by various mammalian cells, especially cancer cells and host cells.^1^ EVs released from cancer cells migrate to blood vessels and flow into various biological fluids, including blood and urine.^2^ Previous experiments have investigated and established that EVs and their available cargoes, including proteins and miRNAs, found in these biological fluids, are important biomarkers for cancer diagnosis.^3^ EVs have been increasingly recognized as promising circulating biomarkers of disease for liquid biopsy. Previous studies have identified plasma EVs as useful markers for various cancer diseases, including lung, pancreatic and breast.^4–7^ So far, membrane proteins such as CD91, CD317, and EGFR have been suggested as potential EV markers of human non-small cell lung cancer.^8–10^ In addition, human cancer-derived cell lines provide to research an almost unlimited and self-replicating source of tumoral cells.^11^ The A549 cells are categorized as a non-small-cell lung carcinoma, which tends to be less aggressive and spread less quickly than small cell lung carcinoma but proves to be more common, accounting for 85–88% of all cases of lung cancer.^12^ The A549 cell line is widely used as a model of lung adenocarcinoma and an *in vitro* model for type II pulmonary epithelial cells.^13^

In the past decade, liquid chromatography-mass spectrometry (LC-MS) is commonly used for untargeted strategy analyses.^14^ The employment of untargeted strategy has led to new biomarker discoveries and a better mechanistic understanding of diseases with applications in precision medicine.^14^ However, many major challenges, such as compound identification have been poor and left an overwhelming number of unidentified peaks.^14^ Recent developments in mass spectrometry have introduced clinical proteomics to the forefront of disease diagnosis, offering reliable, robust, and efficient analytical methods for biomarker discovery and monitoring.^15^ Matrix-assisted laser desorption/ionization-time of flight mass spectrometry (MALDI-TOF MS), which is one of the most powerful tools, has been widely applied to analyze biological samples.^16,17^ Lin and co-workers reported EVs proteome profiling method by using MALDI-TOF MS from the protein extracts.^18^ The results showed the potentials of the MS-based technique in EVs analysis. Stübiger *et al.* used MALDI-TOF MS to analyze the protein profiling of chemoresistance in EVs of cancer cells culture supernatant.^19^ Later, Zhu *et al.* directly detected EVs entities rather than protein extracts.^20^ However, the success of MALDI-TOF MS analyses is highly dependent on the quality of the sample.^21^ For example, contamination of the peptide digest sample with significant levels of detergents, buffer salts, metals, or organic modifiers may greatly inhibit peptide ionization in the MALDI source.^21^ In addition, what remains unclear is whether and how the cell culture conditions, as well as the methods of isolation and storage of EVs would influence the results obtained from MALDI-TOF MS.

Here we described an integrated method involving the EVs separation from the conditioned cell culture medium and MALDI-TOF MS to acquire the protein markers from a human lung carcinoma cell line (A549). We optimized the conditions of cell culture medium components, the procedures of EVs extraction, storage, and MS detection. Among them, cell culture conditions, matrix selection, and EVs storage play a critical role for MS-based EVs analysis from cell samples.

## Experimental

### Cell culture

A549 cells were obtained from Stem Cell Bank, Chinese Academy of Sciences (Shanghai, China) and cultured with a conditioned cell culture medium in a humidified atmosphere in an incubator (Heracell 150i, Thermo Fisher Scientific Inc., MA, USA) with 5% CO_2_ at 37 °C. Roswell Park Memorial Institute (RPMI) 1640 medium, fetal bovine serum (FBS), and 1% penicillin–streptomycin (PS) were obtained from Gibco Life Technologies (CA, USA). The EVs-depleted FBS was purchased from XP Biomed Ltd. (Shanghai, China).

### EVs isolation and characterization

#### EVs isolation

**Fig. 1:**
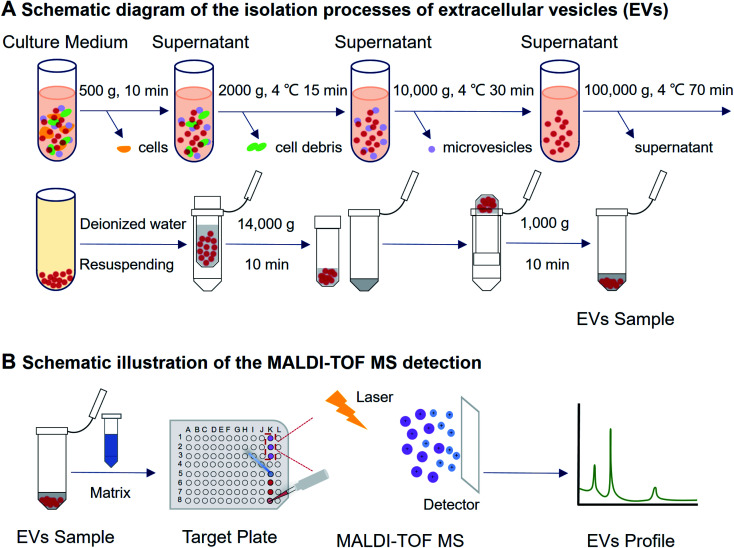
Schematic illustration on the experimental procedure of EVs isolation and MALDI-TOF MS detection. EVs were collected from cell culture medium by using differential ultracentrifugation combined with ultrafiltration, then the mixed sample and matrix were added on a MALDI target plate. With the assistance of a laser energy absorption matrix and pulsed laser irradiation, the protein profile of EVs was generated within a few minutes.

With the high sensitivity of MALDI-TOF MS analysis, it is necessary to isolate EVs with high purity and high concentration. In the experiments, differential ultracentrifugation combined with ultrafiltration was used for EVs isolation instead of using commercially available isolation kits.^22,23^ Briefly, the conditioned cell culture medium was collected after centrifugation of the cell culturing medium at 500 × *g* for 10 minutes (Fig. 1). To avoid cell debris, the supernatant was then centrifuged using 2000 × *g* for 15 minutes at 4 °C. The supernatant was then purified at 10 000 × *g* for 30 minutes at 4 °C. The medium was extracted and further purified at 100 000 × *g* for 70 minutes at 4 °C in an ultracentrifuge (JXN-30, Beckman Coulter, IN, USA). The EVs pellet was resuspended in 100 μL deionized water (Watsons, Hong Kong, China). Amicon® Ultra 100 K filter devices (Merck KGaA, Darmstadt, Germany) were used for further concentration. The sample was concentrated 5-fold by centrifugation at 14 000 × *g* for 10 minutes. The device was turned upside down in a new tube and centrifuged at 1000 × *g* for 1 minute to recover the concentrate.

#### Transmission electron microscopy (TEM)

The morphology of EVs secreted from A549 cells was characterized with a transmission electron microscope (TEM). Briefly, 20 μL of EVs sample was dropped on formvar–carbon coated electron microscopy grids (Beijing Zhongjingkeyi Technology Co., Ltd, Beijing, China) for 1 min and then dried with filter paper. Then EVs were negatively stained with 10 μL 2% (w/v) uranyl acetate (ACMEC Biochemical, Shanghai, China) for 5 min and dried with filter paper. A Tecnai G2 Spirit Twin TEM (FEI, OR, USA) was used to measure EVs at 80 kV.

#### Nanoparticle tracking analysis (NTA)

Furthermore, the EVs' size and concentration were evaluated by a ZetaView PMX 110 NTA instrument (Particle Metrix GmbH, Meerbusch, Germany) with three independent tests. Commercial fluorescent nanoparticles calibrated the NTA analyzer with a diameter of 200 nm before each test at room temperature.

#### Western blot analysis

A549 cells and its EVs were homogenized in lysis buffer (Beyotime Biotechnology, Shanghai, China) respectively, and centrifuged at 13 000 × *g* for 10 minutes at 4 °C to generate a supernatant containing the extracted protein. The protein concentration was measured using a bicinchoninic acid (BCA) protein assay kit (KeyGEN, Nanjing, China). Equal amounts of proteins were separated by SDS-polyacrylamide gel electrophoresis (SDS-PAGE) and transferred onto a polyvinylidene difluoride membrane (PVDF, EMD MilliporeSigma, MA, USA). After blocking with blocking buffer, the blots were incubated with antibodies specific to CD81 (Santa Cruz Biotechnology, Inc., CA, USA) and GAPDH (Proteintech Group Inc., IL, USA) overnight at 4 °C. The membrane was then washed three times in tris-buffered saline with Tween-20 (TBST), and was incubated with goat anti-mouse secondary antibody (Proteintech Group Inc., IL, USA) for 2 hours at room temperature. The immunoreactive proteins were visualized using an enhanced chemiluminescence kit (ABclonal Technology Co., Ltd., Wuhan, China) and an UVP Chemstudio touch 815 (Analytik Jena GmbH, Jena, Germany).

### Effects of different conditions on protein analysis of EVs by MALDI-TOF MS

#### Effects of the medium

To test whether the cell culture medium components influenced EVs detection and cell viability, the A549 cells were cultured in 2% FBS, 2% EVs-depleted FBS, 10% FBS, and 10% EVs-depleted FBS for 2 days, respectively. The cell culture medium and the supernatant of these cells were collected separately for EVs isolation and MALDI-TOF MS analysis.

#### Effects of the matrix

Since the matrix is an essential factor affecting the measurement of MALDI-TOF MS, especially for the micro- and nano-size biological samples. We explored whether commonly used matrices provide satisfactory detection reproducibility for biological samples of a broad mass range.^24^ Trifluoroacetic acid and the MALDI matrix 2,5-dihydroxybenzoic acid (DHB) and 3,5-dimethoxy-4-hydroxycinnamic acid (SA) were purchased from Sigma-Aldrich (St. Louis, MO, USA). All chemicals were purchased at the analytical purity grade available and were used without further purification.

#### Effects of EVs' storage

To test whether storage conditions affect EVs' protein profiling, we exposed the EVs sample to 4 °C for a week and then using MALDI-TOF MS analysis.

### MALDI-TOF MS analysis of EVs protein profiling

The sample preparation and MALDI-TOF MS analytical procedures were the same as reported by Zhu *et al.*^20^ Briefly, the obtained EVs were deposited on the MALDI target plate (1 μL for each spot). The matrix was dropped onto the dried sample spots to overlay the EVs (1 μL for each spot). After that, the target plate was loaded into a MALDI-TOF MS for measurement. With a laser energy absorption matrix's assistance, a mass spectrometry fingerprint was generated within a few minutes under pulsed laser irradiation. Bruker UltrafleXtreme MALDI TOF/TOF (MA, USA) MS was utilized for protein marker analysis. All measurements were conducted under positive linear mode with 20 kV accelerating voltage. Instrumental parameters were set as mass range *m*/*z* 2000–50 000, laser intensity 70%, laser attenuator with 30% offset and 40% range, 300 laser shots accumulation for each spot, 20.0 Hz laser frequency, 20× detector gain, suppress up to 1000 Da, 350 ns pulsed ion extraction.

### Statistical analysis

The results were presented as mean ± standard deviation (S.D.). The unpaired *t*-test was performed using Prism 8 (GraphPad Software, CA, USA). All data were obtained from three individual experiments.

## Results and discussion

### Characterization of EVs derived from A549 cells

**Fig. 2:**
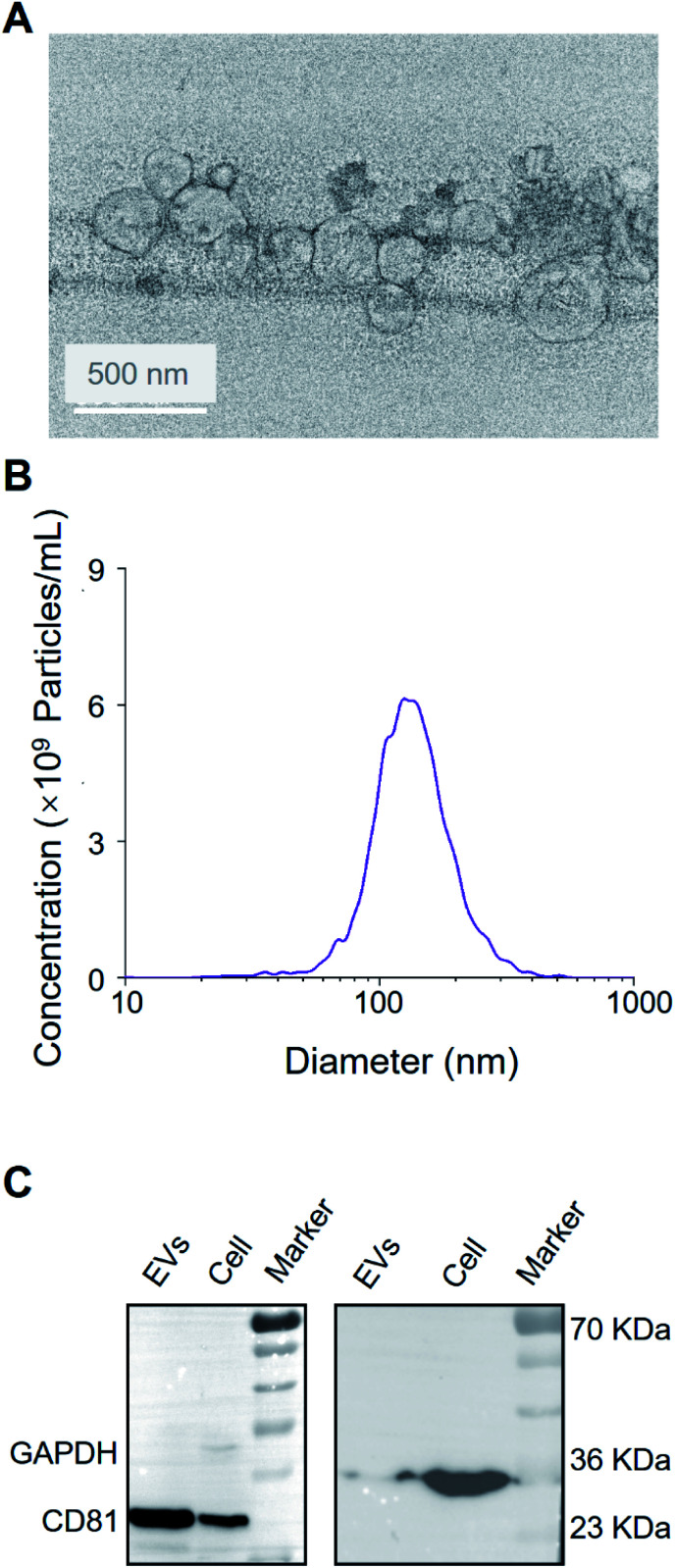
Characterization of extracellular vesicles derived from A549 cells. (A) Transmission electron microscopy of EVs. Scale bar: 50 nm. (B) The size and concentration of A549-EVs using nanoparticle tracking analysis. (C) Western blot analysis of A549 cells and A549-EVs.

As shown in Fig. 2A and B, the vesicles showed the characteristic cup-shaped typical topography with the size of 30–200 nm.^25,26^ Western blot showed CD81 and the endogenous control GAPDH blotting on A549 EV lysate and cell lysate (see Fig. 2C), which is consistent with previous works.^27^

### Identification of the protein profiling of EVs by MALDI-TOF MS

**Fig. 3:**
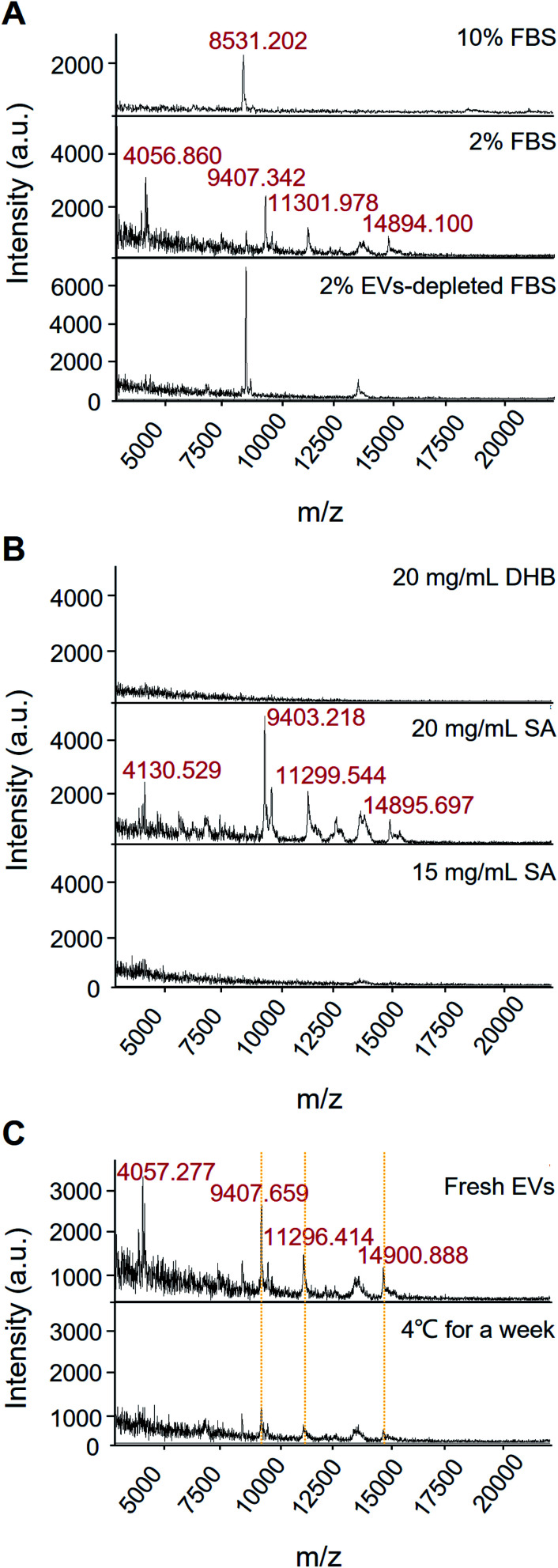
Effects of different conditions on protein analysis of EVs by MALDI-TOF MS. (A) Fetal bovine serum. (B) Matrix selection. (C) Storage conditions.

EVs from the growth medium without culturing cells have three protein peaks (*m*/*z* 6822.716, *m*/*z* 8535.526, and *m*/*z* 14 901.095) (see Fig. S1,† “Culture medium” group). EVs from A549 cells cultured in 10% FBS and 10% EVs-depleted FBS cell culture medium have two protein peaks, which are similar to the control group, but not the specific peaks for the EVs of the A549 cells. It indicates that the high concentration of FBS condition may influence the specific protein profiling of EVs. Compared with 10% FBS, various protein fingerprints were detected upon the 2% FBS cell culture without significant cancer cell proliferation effects (see Fig. 3A and S2†). Conditioned cell culture medium with normal serum can generate more signals than EVs-depleted serum. In Fig. 3A, the EVs isolated in the 2% EVs-depleted medium did not provide any specific signal. Therefore, EVs in 2% FBS serum contribute more to providing the extracellular matrix for cell growth but not affecting EVs' characteristic signals secreted by cancer cells.

The selection of matrix is of importance in MALDI-MS analysis. As shown in Fig. 3B, 15 mg mL^−1^ SA and 20 mg mL^−1^ DHB only got limited signal, but the condition of 20 mg mL^−1^ SA can acquire high-quality mass spectrum. Besides, we also have found that the 20 mg mL^−1^ SA condition contributes the satisfactory reproducibility for cell sample detection.

Moreover, most of the existing analytical tools need to use fresh EVs. Since it takes time from EVs' isolation to detection, the target signal could be lost if there is no suitable storage condition or detection method. It has been reported that low-temperature storage can cause an increase in the size of EVs and destabilize protein content.^28^ These proteins represent EVs leakage or dissociation of loosely bound “peri-exosomal” proteins, suggesting that distinct protein groups leak from EVs at different storage temperatures.^22^ Therefore, EVs should be analysed immediately after isolation for preservation of the EVs protein content and representative functional analysis.^22^ Our results showed that after storing the sample at 4 °C for a week, the corresponding fingerprint of the EVs can still be detected with down-regulated intensity, suggesting that the EVs samples may be degraded at low storage temperature for a short period (Fig. 3C). The results also demonstrated that MALDI-TOF MS is a rapid and less invasive method for identifying EVs quality, which is beneficial to the multiple detection and analysis of precious clinical samples.

In brief, A549 cells were cultured in a medium with 2% FBS for 48 hours. Then, EVs were isolated by differential ultracentrifugation combined with ultrafiltration. 1.0 μL of concentrated EVs was mixed with 1.0 μL of SA matrix (20 mg mL^−1^) and then exposited on the target plate to form co-crystals after air-drying, and loaded into a MALDI-TOF MS for protein marker detection. Among them, cell culture conditions, matrix selection, and EVs storage play a critical role for MS-based EVs analysis from cell samples.

### Typical protein analysis

According to the top-down and bottom-up proteomic analysis from Zhu *et al.*, two typical proteins were identified.^20^ RPS27A: UniProt accession number P62979, MALDI-TOF MS peak: 9405 ± 2 Da *m*/*z*; adjusted precursor mass 9405.88 Da, precursor mass 9405.90 Da, number of matched fragment ions 32, *E* value 5.52 × 10^−25^, *P* value 6.19 × 10^−33^. RPS27A (ribosomal protein; UniProt accession number P62979 [*Homo sapiens* (human)]) (top-down analysis: G.AKKRKKKSYTTPKKNKHKRKKVKLAVLKYYKVDENGKISRLRRECPSDECGAGVFMASHFDRHYCGKCCLTYCFNKPED; bottom-up analysis: MQIFVKTLTGKTITLEVEPSDTIENVKAKIQDKEGIPPDQQRLIFAGKQLEDGRTLSDYNIQKESTLHLVLRLRGGAKKRKKKSYTTPKKNKHKRKKVKLAVLKYYKVDENGKISRLRRECPSDECGAGVFMASHFDRHY CGKCCLTYCF NKPEDK) was detected from EVs of human lung carcinoma cells, which is associated with the cancer cell migration and invasion. RPS27A also plays an essential role in mRNA translation and ribosome assembly for differentiation of cancer cells. Up-regulated or over-expressed RPS27A was displayed in advanced cancer phases.^29,30^ The ubiquitin hybrid gene Uba80 are overexpressed during the cell apoptosis progression by fusing with RPS27A.^31^ In addition, S100A10_S100 calcium binding protein A10 (UniProt accession number P60903 [*Homo sapiens* (human)]) (top-down analysis: M.PSQMEHAMETMMFTFHKFAGDKGYLTKEDLRVLMEKEFPGFLENQKDPLAVDKIMKDLDQCRDGKVGFQSFFSLIAGLTIACNDYFVVHMKQKGKK; bottom-up analysis: MPSQMEHAMETMMFTFHKFAGDKGYLTKEDLRVLMEKEFPGFLENQKDPLAVDKIMKDLDQCRDGKVGFQSFFSLIAGLT IACNDYFVVHMKQKGKK), as a marker for melanoma diagnosis, was also read.^32^ S100A10_S100: UniProt accession number P60903; MALDI-TOF MS peak: 11 299 ± 2 Da *m*/*z*; adjusted precursor mass 11 299.47 Da, precursor mass 11 299.46 Da, number of matched fragment ions 25, *E* value 3.35 × 10^−25^, *P* value 1.23 × 10^−32^. More importantly, up-regulated S100A10 was found in various cancers, implicating that it has potential as a predictive molecule to pharmaceutical effects of anticancer drugs and prognostic markers.^33^

## Conclusions

Recently, EVs are increasingly recognized as the important vehicles of intercellular communication and circulating biomarkers for disease diagnoses and prognosis. In this study, we investigated and optimized the critical experimental conditions for EVs' protein identification in human lung carcinoma cell line by MALDI-TOF MS. Among them, cell culture conditions, matrix selection, and EVs storage play a critical role for MS-based EVs analysis from cell samples. Moreover, we identified two proteins that have been previously implicated as having a role in lung carcinogenesis and may serve as diagnostic markers of lung cancer. In the future, MALDI-TOF MS-based strategy for identifying protein markers shows a great potential to tumor procession, treatment, and pharmacological effect analysis, especially for rapid detection of precancerous disease or lesion without biopsy to evaluating the novel diagnosis method and chemotherapy monitoring.

To summarize, the combination of differential ultracentrifugation and MALDI-TOF MS serves as a rapid and less invasive method for identification of EVs quality and tracking of targeted protein marker. Further extension approaches are needed to explore the tumor progression by EVs-regulated interaction. Therefore, this study provides a solid foundation for the future development of a platform that utilizes EVs as a targeted protein phenotypic tool in clinical studies.

## Conflicts of interest

There are no conflicts to declare.

## Author contributions

H. Y., Z. Y. and C. Z. conceived the study. H. Y. and Q. L. supervised the project. Z. Y., C. Z. and H. Y. designed the experiments. Z. Y., C. Z., S. H., H. Z., W. L., and R. Z. performed the experiments. Z. Y. and C. Z. wrote the manuscript. C. Z., Q. L. and H. Y. supervised and edited the manuscripts.

## Supplementary Material

RA-011-D1RA04305F-s001
