# Supplementary material for: MALDI-MS-based biomarker analysis of extracellular vesicles from human lung carcinoma cells
Source: RSC Adv. 2021 Jul 21;11(41):25375–80. doi: 10.1039/d1ra04305f (PMC9037017; doi:10.1039/d1ra04305f)
Supplement: RA-011-D1RA04305F-s001 [file RA-011-D1RA04305F-s001.pdf]

Electronic Supplementary Material (ESI) for RSC Advances.
This journal is © The Royal Society of Chemistry 2021

Supporting Information

MALDI-MS-based biomarker analysis of extracellular vesicles from human lung carcinoma cells

Zitong Yu,[a] Chao Zhao,[a] Shi Hu,[a] Huitao Zhang,[b] Wenbo Li,[a] Renjie Zhang,[a] Qian Luo *[a] and Hui Yang *[ac]

a. Bionic Sensing and Intelligence Center, Institute of Biomedical and Health Engineering, Shenzhen Institute of Advanced Technology, Chinese Academy of Sciences, Shenzhen 518055, China

b. Research Center for Medical Artificial Intelligencer, Institute of Biomedical and Health Engineering, Shenzhen Institute of Advanced Technology, Chinese Academy of Sciences, Shenzhen 518055, China

c. CAS Key Laboratory of Health Informatics, Shenzhen Institute of Advanced Technology, Chinese Academy of Sciences, Shenzhen 518055, China

* Corresponding Authors

Qian Luo. E-mail: qian.luo@siat.ac.cn.

Hui Yang. E-mail: hui.yang@siat.ac.cn.

Section S1. Effects of cell culture medium on protein analysis of EVs

EVs were isolated from different conditions using the ultracentrifugation method and then analyzed by MALDI-TOF MS. The results showed that EVs from conditioned cell culture medium without cell culture have three protein peaks (m/z 6822.716, m/z 8535.526, and m/z 14901.095). However, EVs from A549 cells cultured in FBS free culture medium have no pronounced protein peak due to its low EVs concentration (2.7 × 10^7 EVs/mL, see Table S1), indicating an aqueous solution containing at least 10^8 EVs/ mL was able to produce a high-quality mass spectrum. Besides, EVs from A549 cells cultured in 10% FBS and 10% EVs-depleted FBS cell culture medium have two protein peaks, which are similar to the control group, not the specific peaks for A549 cells' EVs. Therefore, it is speculated that a high FBS concentration may influence the specific protein profiling analysis of cells.

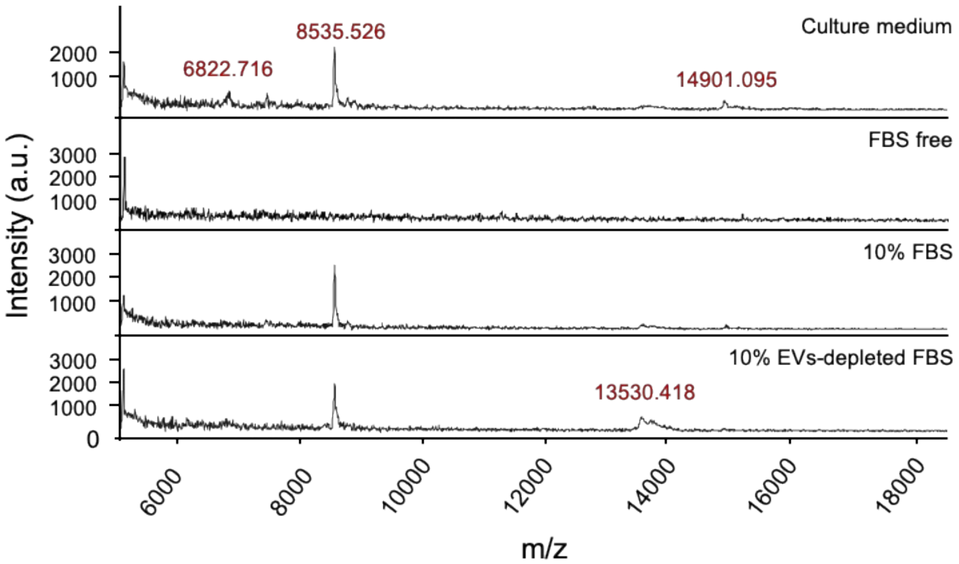

Fig. S1 Effects of fetal bovine serum on protein analysis of EVs by MALDI-TOF MS. EVs were isolated from following conditions: Culture medium, conditioned cell culture medium as a control group; FBS free, A549 cells were cultured in FBS-free culture medium for 48 h; 10% FBS, A549 cells were cultured in 10% FBS culture medium for 48 h;10% EVs-depleted FBS, A549 cells were cultured in 10% EVs-depleted FBS culture medium for 48 h, respectively.

Section S2. Cell viability assay

The viability of cells was measured using a Cell Counting Kit-8 (CCK-8) assay (Beyotime Biotechnology, Shanghai, China). In brief, 2×10^3 A549 cells were cultured in different culture conditions for 48 hours, and an equal volume of the medium was used as a blank control. After 24 h and 48 h, 10% CCK-8 agent was added to the medium and then cells were incubated for another 3 h at 37 °C in the dark. Absorbance at 450 nm was measured with Multiskan FC Microplate Reader (Thermo Fisher Scientific Inc., USA). The results showed that A549 cells could proliferate stably in the following conditions for 48 hours without significant difference, indicating their good cell viability.

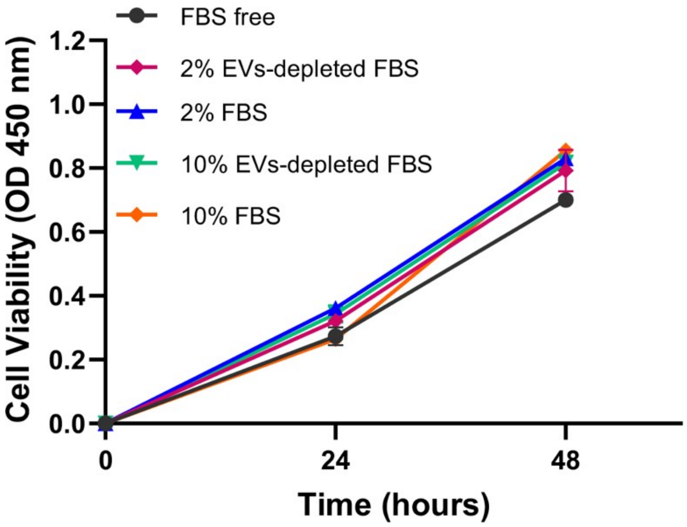

Fig. S2 Cell viability of the A549 cells examined by CCK-8 assay. A549 cells were cultured in following conditions: FBS free (black round points), FBS free culture medium; 2% EVs-depleted FBS (rose red rhomboid points), 2% EVs-depleted FBS culture medium; 2% FBS (blue triangle points), 2% FBS culture medium; 10% EVs-depleted FBS (green inverted triangle points), 10% EVs-depleted FBS culture medium; 10% FBS (orange diamond points), 10% FBS culture medium, respectively. All data are mean ± standard deviation from three independent experiments.

Table S1. The number of A549 cells and the amount of EVs isolated after 48 hours of culture under different culture conditions

A549 cells were cultured in the following conditions for 48 hours, and the EVs were extracted by ultracentrifugation combined with ultrafiltration. The concentration of cells and EVs were detected using Luna-II cell count (Logos Biosystems Inc., Gyeonggi-do, South Korea) and nano-flow cytometry (NanoFCM Co., Ltd, Xiamen, China), respectively.

Group	Cell concentration ($\times 10^6$ cells/mL)	EVs concentration ($\times 10^8$ EVs/mL)
FBS free	5.35 ± 0.33	0.31 ± 0.04
2% EVs-depleted FBS	7.51 ± 1.07	2.52 ± 0.15
2% FBS	8.67 ± 0.84	6.22 ± 1.32
10% EVs-depleted FBS	10.39 ± 1.15	3.73 ± 3.40
10% FBS	9.23 ± 0.53	53.5 ± 2.25

Although A549 cells can proliferate stably for at least 48 hours in an FBS-free medium, few EVs were isolated from the same volume supernatant, which was not enough for MALDI-TOF MS analysis. It is necessary to increase the cell culture volume by 2-10 times to reach the limit of MALDI-MS analysis detection (5×10^7 EVs/mL),[1] which is materials- and time-consuming. Furthermore, it is not clear whether the FBS-free culture will change the cell state. According to our group's preliminary research results, the cell status is positively correlated with its ability to secrete EVs (data not shown). The EVs-depleted serum can minimize the impact of background interference caused by normal serum. However, the results showed that MALDI-MS could not detect any characteristic signals when A549 cells were treated by EVs-depleted cell culture conditions. Besides, EVs from A549 cells that were cultured in 2% normal serum conditions can detect characteristic signals compared with the control group. Therefore, we conclude that EVs isolated from A549 cells under the condition of 2% FBS conditioned cell culture medium can be analyzed by MALDI-TOF MS.

REFERENCES

1. Zhu, Y.; Pick, H.; Gasilova, N.; Li, X.; Lin, T.-E.; Laeubli, H. P.; Zippelius, A.; Ho, P.-C.; Girault, H. H., MALDI detection of exosomes: A potential tool for cancer studies. *Chem* **2019**, *5*, 1318-1336.
